# Supplementary material for: A high-resolution mRNA expression time course of embryonic development in zebrafish
Source: eLife. 2017 Nov 16;6:e30860. doi: 10.7554/eLife.30860 (PMC5690287; doi:10.7554/eLife.30860)
Supplement: Supplementary file 6. [file elife-30860-supp6.zip › biolayout-clusters-files/Cluster151-genes.html]

Cluster151


# Cluster151: Genes

| Ensembl ID | Gene Name | Chr | Start | End | Biotype |
| --- | --- | --- | --- | --- | --- |
| ENSDARG00000077860 | ankhd1 | 21 | 26355058 | 26446542 | protein\_coding |
| ENSDARG00000060320 | dcaf5 | 13 | 36283174 | 36313135 | protein\_coding |
| ENSDARG00000043031 | ints10 | 14 | 693733 | 711671 | protein\_coding |
| ENSDARG00000020840 | mcrs1 | 11 | 3299121 | 3313183 | protein\_coding |
| ENSDARG00000091512 | nfrkb | 21 | 25209594 | 25235811 | protein\_coding |
| ENSDARG00000077751 | nol9 | 23 | 25028410 | 25044255 | protein\_coding |
| ENSDARG00000095538 | senp3a | 5 | 23716357 | 23727339 | protein\_coding |
| ENSDARG00000102923 | senp6a | 20 | 679666 | 691744 | protein\_coding |
| ENSDARG00000074298 | si:ch73-266f23.1 | 4 | 71811085 | 71920152 | protein\_coding |
